# Case Report: Novel *CA12* Homozygous Variant Causing Isolated Hyperchloridrosis in a Chinese Child With Hyponatremia

**DOI:** 10.3389/fped.2022.820707

**Published:** 2022-03-14

**Authors:** Meigui Han, Min Peng, Ziming Han, Xiaojuan Zhu, Qian Huang, Weiyue Gu, Yong Guo

**Affiliations:** ^1^Department of Pediatrics, The First Affiliated Hospital of Xinxiang Medical University, Xinxiang, China; ^2^Chigene Translational Medicine Research Center Co., Ltd., Beijing, China; ^3^Department of Physiology and Pathophysiology, Xinxiang Medical University, Xinxiang, China

**Keywords:** isolated hyperchloridrosis, *CA12* gene, dehydration, hyponatremia, hypochloremia

## Abstract

Isolated hyperchloridrosis (HYCHL; OMIM 143860) is a rare autosomal recessive disorder caused by biallelic mutations in the *carbonic anhydrase 12 (CA12; OMIM 603263)* gene, which is characterized by abnormally high levels of salt in sweat that can lead to dehydration associated with low levels of sodium in the blood. To date, only four variants of the *CA12* gene have been identified to be associated with HYCHL. Here, we presented a rare Chinese case of HYCHL in an infant with decreased food intake, mild diarrhea, severe dehydration, and hypovolemic shock who was hospitalized in our department three times. Laboratory tests showed hyponatremia and hypochloremia. Because of recurrent attacks, whole-exome sequencing (WES) was performed and revealed a novel homozygous missense variant c.763A>C (p.Thr255Pro) in the *CA12* gene (NM_001218.5). In total 0.9% sodium chloride (NaCl) solution was orally administered until 1 year and 6 months of age. Followed up to 3 years of age, the patient showed good growth and development without similar manifestations. This study reported a novel *CA12* gene mutation leading to HYCHL for the first time in China, which enriched the genotype of HYCHL and emphasized the early suspicion and identification of the rare condition to adequate treatment.

## Introduction

Isolated hyperchloridrosis is a rare autosomal recessive condition caused by homozygous or compound heterozygous mutations in the *CA12* gene on chromosome 15q22.2, and its prevalence is unknown (1–3). *CA12* gene mutations can reduce the activity of carbonic anhydrase XII, cause changes in chloride ion-mediated negative feedback regulation of the enzyme, which result in excessive chloride secretion in sweat and clinical manifestations of hyponatremic hypochloraemic dehydration (4). Only three pieces of literature describe this disease caused by *CA12* gene mutations. This study describes a Chinese boy who was admitted at 8 months of age. Before admission, the patient had decreased food intake, mild diarrhea, and severe dehydration, including one accompanied by hypovolemic shock. Laboratory tests showed hyponatremia and hypochloremia. Electrolyte imbalance was quickly corrected after 0.9% NaCl supplementation. The patient was hospitalized three times due to recurrent hyponatremia and hypochloremic dehydration. Whole-exome sequencing (WES) identified in the child a homozygous pathogenic variant in *CA12* (c.763A>C, p.Thr255Pro), allowing the diagnosis of hyperchloridrosis (HYCHL). We also present a review of relevant literature on the mutation spectrum of the *CA12* gene to help clinicians better recognize HYCHL caused by *CA12* gene mutations. This case demonstrates the importance of genetic tests in children with unusual characteristics, which would allow a timely diagnosis and adequate treatment.

## Case Description

An 8-month-old boy was admitted to the pediatric intensive care unit of our hospital on May 18, 2019, due to “little food intake for 2 days”. He had a poor mental response, severe dehydration, increased heart rate of 200 beats/min, low blood pressure (60/30 mmHg), and prolonged capillary filling time (RT>5s). His blood biochemistry showed Na^+^ 117 mmol/L, Cl^−^ 83 mmol/L, K^+^ 5.8 mmol/L, HCO^3−^ 6.9 mmol/L. Brain MR plain scans and MR enhancements showed no abnormality. Chest CT showed patchy, increased density shadows in the upper lobe of the left lung, suggesting the possibility of left lung infection. The abdominal color ultrasound did not detect any abnormal changes. The child was diagnosed with hyponatremia and hypochloremic dehydration with severe acidosis and hypovolemic shock. The patient was given isotonic sodium-containing solution for volume expansion and correction of acidosis and other treatments. Reexamination of blood biochemistry after one day showed Na^+^ 133 mmol/L, Cl^−^ 95.5 mmol/L, HCO^3−^ 32.3 mmol/L. The dehydration was corrected and the mental response improved. The patient was discharged on the 9th day after hospitalization. The patient was admitted to the pediatric intensive care unit of our hospital for the second time at an interval of 8 days due to “diarrhea for 1 week”. The patient still had a poor mental response, mild dehydration, heart rate 170 beats/min, but normal blood pressure. His blood biochemistry showed Na^+^ 127 mmol/L, Cl^−^ 87 mmol/L, K^+^ 4.9 mmol/L, HCO^3−^ 14.8 mmol/L. After sodium-containing solution supplementation, the blood biochemistry returned to normal. The patient was discharged 4 days after hospitalization. The patient had similar symptoms again at an interval of 3 days after discharge. Blood biochemistry showed Na^+^ 117 mmol/L, Cl^−^ 81.7 mmol/L, K^+^ 4.0 mmol/L, HCO^3−^ 17.1 mmol/L (Table 1). The blood biochemistry returned to normal after sodium-containing solution supplementation was administered. Due to recurrent hyponatremia and hypochloremic dehydration, we considered inherited metabolic diseases and genetic testing was required to further confirm the diagnosis. Informed consent was obtained from the guardians of the child in this study and the study was approved by the medical ethics committee of the First Affiliated Hospital of Xinxiang Medical University (approval number: EC-021-045).

**Table 1:** Laboratory investigations during the three hospital admissions.

**Measured parameters**	**First admission**	**First treatment**	**Second admission**	**Third admission**	**Normal values**
Serum potassium(K^+^)	5.8 mmol/L	No data	4.9 mmol/L	4.0 mmol/L	3.5–5.0 mmol/L
Serum bicarbonate(HCO^3−^)	6.9 mmol/L	32.3 mmol/L	14.8 mmol/L	17.1 mmol/L	22–25 mmol/L
Serum sodium(Na^+^)	117 mmol/L	133 mmol/L	127 mmol/L	117 mmol/L	135–145 mmol/L
Serum chloride(Cl^−^)	83 mmol/L	95.5 mmol/L	87 mmol/L	81.7 mmol/L	98–106 mmol/L

**Figure 1:**
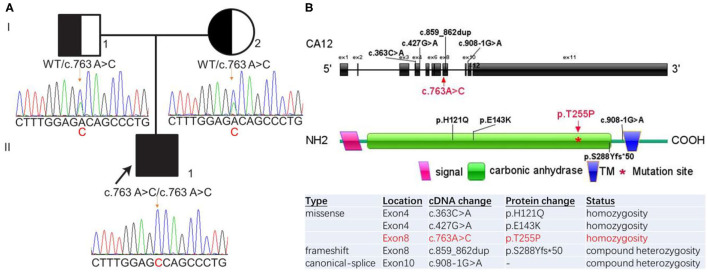
A Chinese family with *CA12* homozygous mutation causing isolated hyperchloridrosis. **(A)** Pedigree and Sanger sequencing electropherograms of the family with *CA12* variants. The patient (II-1) had a homozygous variation c.763A>C (p.T255P) which was inherited from her father (I-1) and mother (I-2), respectively. **(B)** Schematic diagram of the *CA12* gene (NM_001218.5 and NP_001209.1) structure and variants as reported in the literature. The gene structure includes 11 exons (big black box). Previously reported mutations are black-coded. The mutation described in this article is marked red. TM, Transmembrane.

Blood samples of 2–3 ml were collected from the child and his parents and genomic DNA was isolated to carry out WES. DNA fragments were hybridized and captured using the SureSelect human all exon V7 (exome V7) kit (Agilent Technologies, Santa Clara, CA, USA). Sequencing was performed on the Illumina HiSeq 4000 (Illumina, Inc., San Diego, CA, USA) with the 150-bp paired-end read mode to a mean target coverage of 20 × and more than 99% of the target sequence was sequenced. Sequencing raw data were processed by fastq for removing adapters and filtering low-quality reads. The paired-end reads were aligned to the GRCh37/hg19 reference genome using Burrows-Wheeler Aligner (BWA). HiSeq base quality scores were recalibrated using the Genome Analysis Toolkit (GATK) Table Recalibration, and variants were called with GATK Unified Genotyper. Variants were annotated with databases for minor allele frequencies (MAFs) (1,000 genomes, dbSNP, ESP, ExAC, and gnomAD). Pathogenicity prediction was processed based on serial software packages (Provean, Sift, Polypen2_hdiv, Polypen2_hvar, Mutation taster, M-Cap, and Revel software packages for conservative analysis and protein product structure prediction, MaxEntScan, dbscSNV, and GTAG software for predicting the functional change of variants on the splicing sites). Candidate variants were then filtered based on frequency [<0.5% in the 1,000 Genomes, ExAC, and Exome Variant Server (EVS)], inheritance of the disease, effect on protein sequence, and deleteriousness predictions of the variants with the use of OMIM, HGMD and ClinVar databases to the pathogenicity of every variant. WES results revealed the existence of a homozygous missense mutation (c.763A>C, p.Thr255Pro) in exon 8 of *CA12* in the patient, which had not been previously reported. Trio-WES analysis failed to detect other rare variants associated with specific clinical features of this patient. Pedigree validation confirmed that the mutations were inherited from his father and mother, respectively, which was following the rule of autosomal recessive inheritance (Figure 1). The identified variant was Sanger sequenced using a KAPA2G Robμst HotStart PCR Kit (KAPA Biosystems) on a Hema 9600 PCR Thermo Cycler (Zhuhai Hema Medical Instrument Co., Ltd.) with the primers: forward, 5′-AAGGCGCCTGGTTCCCGTG-3′ and reverse, 5′-AACAAGCCCTGCGACCCTCTG-3′ (product size, 663 bp; annealing temperature, 60°C). PCR amplification procedure: pre-denaturation at 95 °C for 3 min; 30 cycles of 95 °C for 30 s, 60 °C for 30 s, 72 °C for 30 s; final extension at 72 °C for 1 min. Then the products were sequenced by ABI 3730XL sequencing apparatus (Applied Biosystems) according to the manufacturer's instructions, and sequences were analyzed *via* DNASTAR. According to American College of Medical Genetics and Genomics guidelines, *CA12* gene mutation c.763A>C is classified as a variant of uncertain significance (VUS) (shreds of evidence: PM1 + PM2 + PP3). The mutation was located in the key functional domain of this gene (moderate pathogenic evidence, PM1). The mutation was absent from controls in the GnomAD database (moderate pathogenic evidence, PM2). Bioinformatic prediction analyses using a variety of statistical methods, including conservation prediction, evolutionary prediction, and splice site effects, indicated that this variant can cause deleterious effects on genes or gene products (possible pathogenic evidence, PP3).

**Figure 2:**
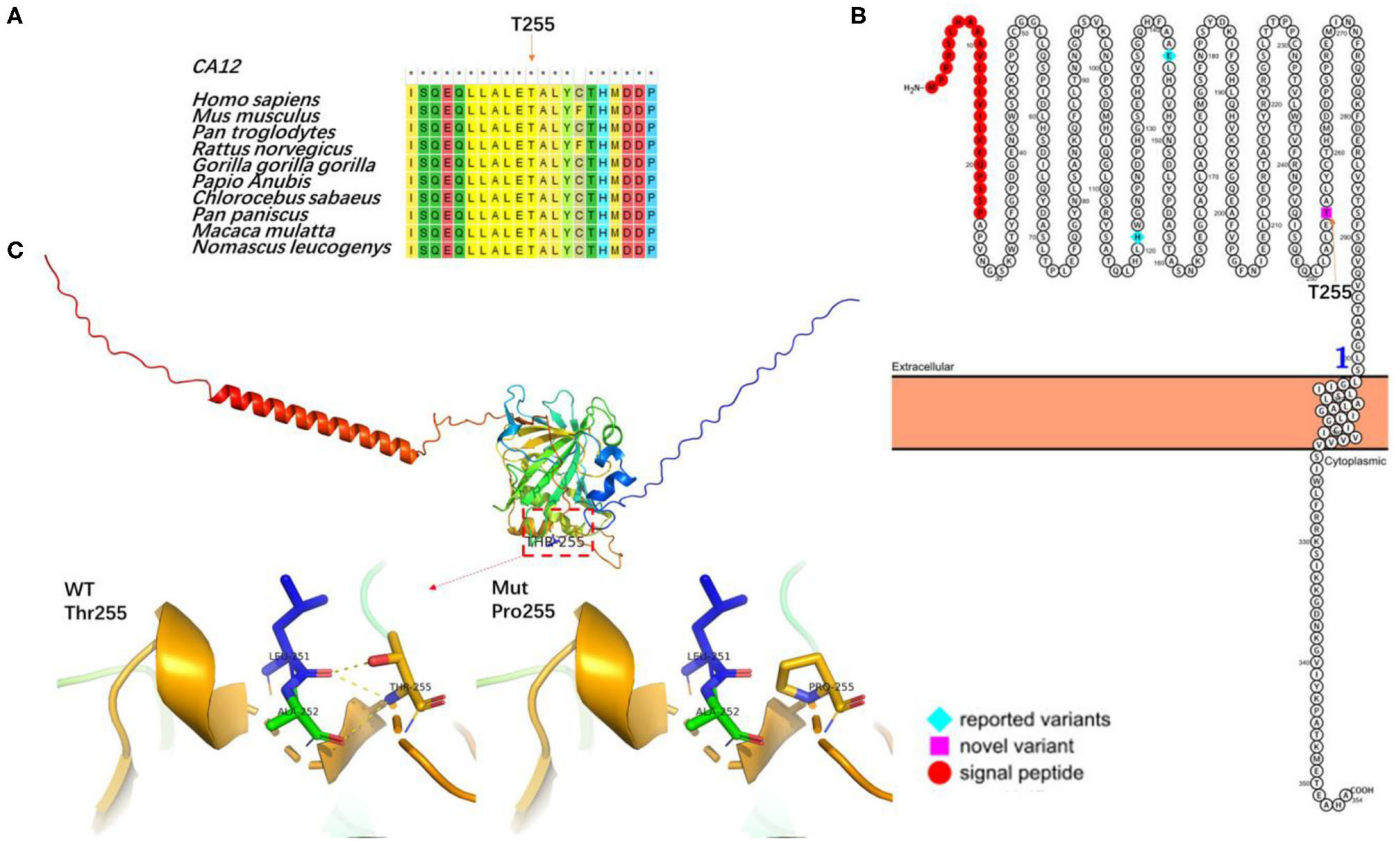
The effect of the mutation on the CA12 protein. **(A)** Sequence alignment of CA12 protein among different species. Asterisks (*) denote highly conserved sites. Conservation analysis by MEGA7 for the p.Thr255Pro residue (marked with an arrow), demonstrating highly conserved. **(B)** Membrane topology plot of *CA12* by PROTTER showing reported variants (blue) and the novel variant (pink). **(C)** Virtual three-dimensional structure models of the mutation analysis were performed with Pymol. The backbone structure of the CA12 protein is illustrated as ribbons and the amino acid residues are highlighted as sticks. The native Thr255 and mutated Pro255 residues are highlighted in dark yellow stick format. Dotted yellow lines represent hydrogen bonds.

To investigate the possible consequences of the novel missense variant c.763A>C of *CA12* gene at the protein level, we performed conservation analysis and further protein structure prediction (Figure 2). Multiple sequence alignment using Mega7 shows the amino acid residue Thr255 of CA12 protein is in the highly conserved region among several species (Figure 2A). Predicted membrane topology model of human CA12 generated in Protter (http://wlab.ethz.ch/protter). The secondary structure of CA12 protein contains a signal peptide, an N-terminal extracellular catalytic domain, a transmembrane a-helix, and a small intracellular C-terminal domain. Thr255 is positioned in the extracellular catalytic domain and adjacent to the membrane-spanning a-helix (Figure 2B). The protein crystal structure of the human *CA12* (AF-O43570-F1) in the Alpha Fold Protein Structure Database was adopted to predict the structure of the wild-type (WT) and mutated domains of *CA12*. Predicted structures of the WT and variant were visualized using the PyMOL software. Hydrogen bonds have a significant effect on the normal space conformation and stability of proteins. Thr255 naturally forms three hydrogen bonds with the side chain of Leu251 and Ala252. The variant p.Thr255Pro replaces a hydrophilic threonine with a hydrophobic proline thereby breaking these hydrogen bonds, indicating that the mutation might have contributed to the abnormal spatial structure and low stability, finally, affecting the functional activity (Figure 2C). In summary, the p.Thr255Pro interferes with the spatial structure of the protein side chains, thereby possibly causing CA12 protein mild loss-of-function. These findings suggest that this novel mutation found in the proband may be the cause of the disease.

A diagnosis of isolated hyperchloridrosis was finally made based on the clinical symptoms and the genetic results. Sodium chloride supplementation continued until 1 year and 6 months of age, and at that time the child was discharged with no similar conditions occurring. During follow-up to 3 years of age, the growth and development were normal, suggesting that the sodium chloride treatment had a positive effect and a good prognosis for isolated hyperchloridrosis.

## Discussion

Hyponatremia, defined as serum sodium less than 135 mmol/L, is the most common disorder of electrolyte homeostasis in clinical practice that occurs in 15–30% of acutely or long-term hospitalized patients and can increase in-patient mortality (5, 6). Hyponatremia is the biochemical manifestation caused by a wide range of clinical conditions. The common causes of hyponatremia in children are excessive salt loss, reduced salt intake, and increased plasma volume (7). Genetic diseases leading to excessive salt loss from sweat glands include renal pseudohypoaldosteronism type I (PHA1) and cystic fibrosis (CF) (8). PHA1 (OMIM 177735) is an autosomal dominant disease characterized by mineralocorticoid resistance, caused by heterozygous mutation in the mineralocorticoid receptor gene (*NR3C2*), presenting in infancy with highly variable degrees of dehydration and dysplasia, and blood biochemical tests suggest hyponatremia, hyperkalemia, metabolic acidosis, elevated levels of plasma renin and aldosterone, and increased sweat chloride (9). Autosomal recessive pseudohypoaldosteronism type I (PHA1B; OMIM 264350), caused by the mutation in any one of the three genes (*SCNN1A, SCNN1B, SCN1G*) encoding the epithelial sodium channel (ENaC), is a similar but more severe disorder with persistence into adulthood. CF (OMIM 219700) is an autosomal recessive disorder caused by a homozygous or compound mutation in the cystic fibrosis conductance regulator gene (*CFTR*), with various clinical symptoms such as bronchiectasis, cholestatic cirrhosis, digestive dysfunction, and vas deferens dysfunction, and involvement of sweat glands will manifest as hyponatremia and increased sweat chloride (10). In this case, the patient's most prominent manifestations were recurrent hyponatremia. WES found that deleterious variants were absent in the disease-causing genes *NR3C2, SCNN1A, SCNN1B, SCNN1G*, and *CFTR* of PHA or CF, which excluded the diagnosis of the above two diseases. A previously unreported variant c.763A>C in exon8 of the *CA12* gene was found in homozygosity in the proband, which may be associated with isolated hyperchloremia. While hyperchloridrosis can occur as one of several features of other conditions, such as cystic fibrosis, people with isolated hyperchloridrosis do not have the additional signs and symptoms of these other conditions.

Isolated hperchloridrosis, first introduced in 2010 by Feldshtein, and also known as carbonic anhydrase XII (CA XII) deficiency, is a rare autosomal recessive condition in which excessive salt wasting in sweat can result in severe infantile hyponatremic dehydration and hyperkalemia. The incidence of isolated hyperchloridrosis has not been reported in China and only three studies have confirmed the disease by genome-wide linkage analysis or WES (1–3). In total, 13 previously published cases were identified in the literature and four variants in the *CA12* gene were reported to account for the disease, including 1frameshift (c.859_860dup), 2 missense (p.E143K and p.H121Q), and 1 canonical splice (c.908-1 G>A). Together with the presented case, 14 cases (9 males and 5 females) and four *CA12* mutations were summarized for analysis (Table 2). Among all the reported cases, most were homozygous and only one was compound heterozygous, with the p.E143K mutation being the most common. Excessive salt in sweat and increased sweat chloride levels are consistent features. Clinical manifestations of HYCHL due to biallelic *CA12* mutations are usually onset in infancy due to poor feeding, failure to thrive and poor weight gain in infancy, intermittent hypotonic dehydration, recurrent hyponatremia, and hyperkalemia. Some patients had respiratory symptoms (mild obstructive airway disease). Our patient had been hospitalized repeatedly with mild gastrointestinal symptoms and mild to severe dehydration. Laboratory tests suggested hyponatremia and hypochloremia, which were consistent with previous literature reports. The presence of elevated chloride in sweat could not be verified without collecting sweat for electrolyte examination.

**Table 2:** Previously reported clinical features of HYCHL due to biallelic *CA12* mutations.

**Year**	**Case**	**Gender**	**Age**	**Ethinic**	**Mutations**	**Clinical feature**	**Sweat test**	**References**
2010	1	Male	ND	Bedouin	c.427G>A/ c.427G>A	Seven individuals with sweat chloride levels substantially above 60 mmol/L, dramatically excessive amounts of visible salt precipitates in sweat (especially in the nape). Five of the seven individuals were hospitalized once before the age of one year for hyponatremic dehydration (mostly following mild gastroenteritis), poor feeding, and slow weight gain, with steatorrhea recorded in two of the cases.	110	Feldshtein et al. (1)
	2	Female	ND	Bedouin	c.427G>A/ c.427G>A		156	
	3	Male	ND	Bedouin	c.427G>A/ c.427G>A		95	
	4	Male	ND	Bedouin	c.427G>A/ c.427G>A		128	
	5	Female	ND	Bedouin	c.427G>A/ c.427G>A		116	
	6	Male	ND	Bedouin	c.427G>A/ c.427G>A		155	
	7	Female	ND	Bedouin	c.427G>A/ c.427G>A		85	
2011	8	Male	1m	Bedouin	c.427G>A/ c.427G>A	Acute gastroenteritis, dehydration, FTT, hyponatremia, hyperkalemia	190	Muhammad et al. (2)
	9	Male	4m	Bedouin	c.427G>A/ c.427G>A	Restlessness, dehydration, FTT, hyponatremia, hyperkalemia	60	
	10	Male	3m	Bedouin	c.427G>A/ c.427G>A	Fever, diarrhea, severe FTT, neonatal urinary tract infection, dehydration, hyponatremia, hyperkalemia	160	
2016	11	Female	25y	America	c.908-1 G>A/c.859_860dup	Elevated sweat chloride, recurrent hyponatremia, infantile FTT, and lung disease (pulmonary exacerbations, Pseudomonas in sputum cultures, and mild but distinct bronchiectasis upon high-resolution chest CT scanning)	99.5	Lee et al. (3)
	12	Female	11y	Omani	c.363 C>A/c.363 C>A	Elevated sweat chloride, recurrent hyponatremia, infantile FTT, and bilateral hyperkeratosis of the heels	130	
	13	Male	6y	Omani	c.363 C>A/c.363 C>A		97	
Current study	14	Male	9m	China	c.763A>C/ c.763A>C	Recurrent hyponatremia, diarrhea	ND	This report

*For each patient, the column shows results at the first checkup. ND, not mentioned; y, years; m, months; d, days; FTT, failure to thrive; Sweat Test: normal <60 mmol/l-Cl^−^*.

*CA12* contains 11 exons that encode carbonic anhydrase XII with 354 amino acids. CA XII is a transmembrane zinc metalloenzyme, which is known to play a central role in regulating many cancers, but its function and mechanism in hyperchloridrosis are rarely discussed. CA XII is involved in the reversible hydration of carbon dioxide and water and regulates the transport of chloride in the tissues, such as sweat glands, kidneys, and intestines, which contribute to the maintenance of extracellular pH. CA XII can control the amount of salt released in sweat by regulating cellular pH in the sweat glands *via* bicarbonate metabolism. CA XII has been identified to be biologically active as an isologous dimer. The active site clefts of CA XII lies on the one face of the dimer, and the C-terminal on the opposite face of the dimer to facilitate membrane interaction. The Zn^2+^ ion, located at the bottom of the active site cleft, is an essential cofactor for carbonic anhydrases and enzymatic biological activity (11). In the extracellular catalytic domain of human CA XII, four direct ligands (the hydroxide ion and three histidine residues His 119, His121, His145) hold the zinc in place by a “second coordination shell” (12). To date, four *CA12* variants have been reported associated with isolated hyperchloridrosis, including p.E143K and p.H121Q in exon4, c.908-1 G>A in exon9 and c.859**_**860dup in exon8. The p.E143K mutation altered sensitivity to anions, affected coordination of the zinc ion within the catalytic domain, prominently inhibited ductal fluid secretion and salivation *in vivo* through inhibition of epithelial AE2, and caused misfolding and mistargeting of *CA12* due to partial degradation and aberrant *CA12* glycosylation (13). The p.H121Q mutation was essential for tetrahedral coordination of the zinc ion in the enzyme active site and generated stable protein but enzyme activity was near complete loss when assayed at physiologic concentrations of extracellular chloride. The c.908-1 G>A and c.859**_**860dup mutations generated aberrant *CA12* transcripts that missing nucleotides to form transmembrane domains and unstable protein products that greatly reduced the amount of CA12 protein (3). In this case, WES and co-segregation analysis revealed a novel homozygous missense mutation (c.763A>C, p.T255P) in *CA12*. A multi-species conservation analysis found that the amino acid residue Thr255 was highly conserved, and *in silico* analysis of the mutation indicated that the variant p.T255P could lead to protein structure changes and maybe damage the protein function.

Previously reported patients with infantile-onset isolated hyperchloridrosis responded well to sodium chloride treatment and the symptoms improved rapidly after treatment with sodium chloride supplementation. Catch-up growth occurred after 1 year of age, with the longest span of achieving normal growth being up to 6 years of age. The sodium chloride supplementation prevents hyponatremic dehydration. These individuals may still experience dangerous hyponatremia when they sweat excessively, for example in warm temperatures or when exercising (4). In this study, the clinical and genetic findings allowed the diagnosis of isolated hyperchloridrosis. In addition, we identified a previously unreported pathogenic variant of the *CA12* gene. To our knowledge, this is the first patient-reported with *CA12*-related isolated hyperchloridrosis in China. He presented hyponatremic hypochloremic dehydration of different severity during 3 hospitalizations, which can be the feature of isolated hyperchloridrosis caused by *CA12* mutation. The symptoms were quickly relieved after sodium chloride supplementation. Sodium chloride supplementation was continued until 1 year and 6 months of age, during which no similar conditions occurred. A follow-up to 3 years of age showed that the growth and development were normal, suggesting that early diagnosis has a good prognosis after appropriate treatment.

## Conclusion

In conclusion, *CA12*-related isolated hyperchloridrosis is rare and difficult to diagnose. Our case revealed that the novel homozygous p.T255P mutation in the *CA12* gene was involved in the development of isolated hyperchloridrosis in the Chinese population and additional experiments are needed to study the underlying mechanisms of *CA12* in isolated hyperchloridrosis. The clinical possibility of *CA12* biallelic mutation associated with isolated hyperchloridrosis should be considered in children with hyponatremic hypochloremic dehydration. In this study, we also emphasized the early identification of this condition, which would prevent complications and adapt the instituted treatments.

## Data Availability Statement

The datasets presented in this article are not readily available because of concerns regarding participant/patient anonymity. Requests to access the datasets should be directed to 021017@xxmu.edu.cn.

## Ethics Statement

The studies involving human participants were reviewed and approved by the Medical Ethics Committee of the First Affiliated Hospital of Xinxiang Medical University (Approval Number: EC-021-045). Written informed consent to participate in this study was provided by the participants' legal guardian/next of kin.

## Author Contributions

MH conceptualized and designed the study, drafted the initial article, and reviewed the article. MP performed the molecular analysis and analyzed the WES data. XZ, QH, and ZH collected the patients' clinical information. YG designed the study and reviewed the article. WG provided technical support for this work. All authors contributed to the article and approved the submitted version.

## Conflict of Interest

MP and WG were employed by Chigene Translational Medicine Research Center Co., Ltd. The remaining authors declare that the research was conducted in the absence of any commercial or financial relationships that could be construed as a potential conflict of interest.

## Publisher's Note

All claims expressed in this article are solely those of the authors and do not necessarily represent those of their affiliated organizations, or those of the publisher, the editors and the reviewers. Any product that may be evaluated in this article, or claim that may be made by its manufacturer, is not guaranteed or endorsed by the publisher.
